# Estimated Incubation Period for Zika Virus Disease 

**DOI:** 10.3201/eid2305.161715

**Published:** 2017-05

**Authors:** Elisabeth R. Krow-Lucal, Brad J. Biggerstaff, J. Erin Staples

**Affiliations:** Centers for Disease Control and Prevention, Atlanta, Georgia, USA (E.R. Krow-Lucal);; Centers for Disease Control and Prevention, Fort Collins, Colorado, USA (E.R. Krow-Lucal, B.J. Biggerstaff, J.E. Staples)

## Abstract

Information about the Zika virus disease incubation period can help identify risk periods and local virus transmission. In 2015–2016, data from 197 symptomatic travelers with recent Zika virus infection indicated an estimated incubation period of 3–14 days. For symptomatic persons with symptoms >2 weeks after travel, transmission might be not travel associated.

Zika virus is a mosquito-borne flavivirus transmitted primarily through the bite of infected *Aedes* spp.mosquitoes. Transmission can also occur through occupational laboratory exposure and by intrauterine, intrapartum, or sexual routes (*1**–**3*).

In May 2015, Zika virus disease cases were identified in Brazil, representing the first local transmission in the Americas (*4*). Subsequently, Zika virus spread rapidly, resulting in >463,000 suspected and laboratory-confirmed cases in the Americas as of June 30, 2016 (*5*). This rapid expansion highlighted key knowledge gaps, including incubation period. Characterizing the incubation period for Zika virus is needed for defining periods of risk and identifying local virus transmission. To estimate the incubation period, we used data from symptomatic persons who had traveled to an area with ongoing Zika virus transmission and for whom laboratory evidence indicated recent infection.

## The Study

We included in our analysis persons for whom samples tested at the Centers for Disease Control and Prevention from January 1, 2015, through June 23, 2016, gave positive results, indicating recent Zika virus infection (defined as Zika virus RNA positivity by real-time reverse transcription or Zika or dengue virus positivity by IgM capture ELISA and confirmed by plaque reduction neutralization test with a Zika virus–specific neutralizing antibody titer >10 and Zika virus titer >4-fold higher than dengue virus titer) (*6*,*7*). We restricted our analysis to persons who were symptomatic, had known symptom onset date (onset of first symptom), had known travel dates from/to the continental United States, and were probably infected through a mosquito bite. We excluded from analysis those for whom disease was congenital or sexually transmitted and those reporting illness onset >2 months after travel (because of the typically shorter incubation periods for other flavivirus diseases).

**Figure 1:**
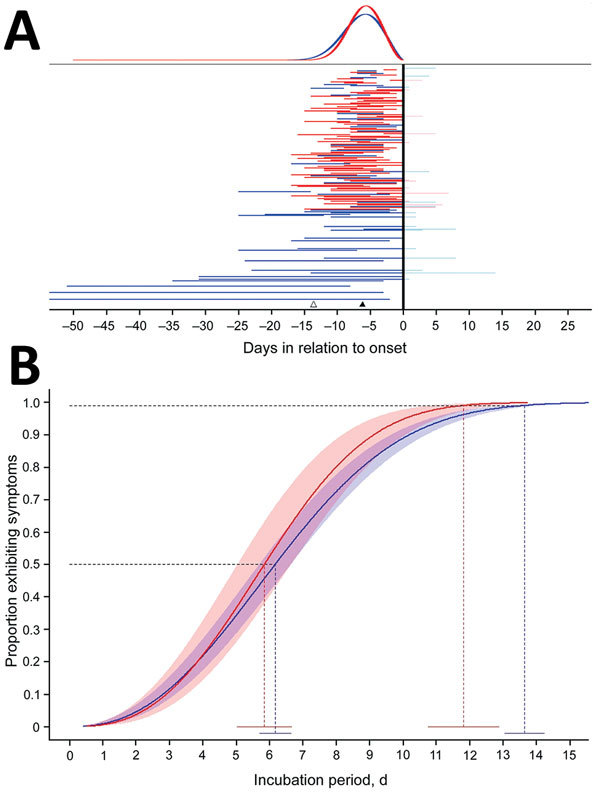
Estimated distribution of incubation period in days since infection for persons with evidence of recent Zika virus disease. A) Representation of individual interval censored travel data based on time of exposure relative to symptom onset (n = 197). Horizontal lines represent exposure times relative to onset. Vertical black line indicates symptom onset; red indicates persons with confirmed Zika virus disease; blue indicates all persons with Zika virus diseases; pink indicates exposure durations after symptom onset; and light blue indicates that these times did not contribute to the analysis. Individual data are sorted from bottom to top by exposure duration; to ease visible interpretation, we truncated long durations. The black triangle marks the estimated median incubation period for all Zika virus disease cases; the white triangle marks the estimated 95th quantile. The top panel shows the fitted Weibull density function; the blue line represents the distribution for all Zika virus disease cases; and the red line represents only those with confirmed Zika virus disease. B) Estimated distribution of time from infection to symptom onset (incubation period) for 197 persons with evidence of recent Zika virus infection (blue) and with confirmed Zika virus disease (red). The heavy line represents the estimated Weibull cumulative distribution function for the incubation period; 95% confidence bands are shown in red and blue shading. The 2 dotted lines represent the 50th and 99th quantiles; blue represents all cases; and red represents confirmed cases only. The solid horizontal line near the *x*-axis gives the point estimates and 95% CIs for the quantiles. Additional quantiles and CIs are shown in Technical Appendix Table 2).

To estimate the incubation period distribution, we first defined the exposure period as either the duration of travel if a person experienced illness after return from travel or the time from beginning of travel to the onset of illness if the traveler became ill during travel (Figure 1, panel A). We then fit various probability distributions in R (https://cran.r-project.org/) by using the dic.fit function in the coarseDataTools package, which uses methods detailed by Reich et al. (*8*). We selected the best model by using the Akaike information criterion. In addition to reporting fitted cumulative distribution function and associated 95% CIs, we reported certain quantiles and means. All analyses were conducted by using R.

For our primary analysis, we used all persons with evidence of a recent Zika virus infection (primary case set). We then performed a secondary analysis of persons with confirmed Zika virus infection and <2 weeks of travel (secondary case set), enabling evaluation of our estimates by using more stringent case definition requirements. A confirmed case of Zika virus disease was illness in a symptomatic person with a sample that was either Zika virus RNA positive or Zika or dengue virus IgM positive with neutralizing antibodies against Zika virus only.

**Figure 2:**
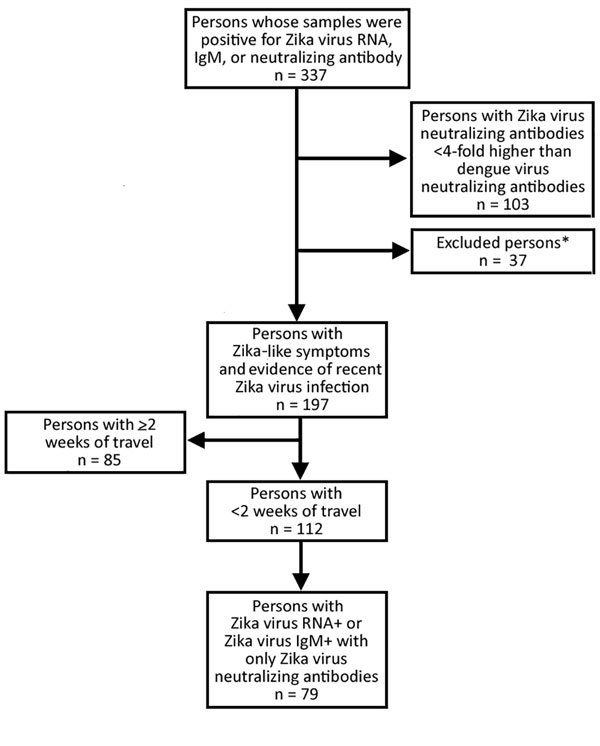
Persons with Zika virus–like symptoms and positive test results for Zika virus infection identified from samples received and tested for Zika virus infection at the Centers for Disease Control and Prevention. *Excluded for being asymptomatic, having congenital infection, having sexually transmitted infection, history of travel originating outside the United States, no date of symptom onset, symptom onset >2 months after travel return.

From January 1, 2015, through June 23, 2016, we identified 337 persons with evidence of recent Zika virus infection. Of these, we excluded 140 (42%) because they did not meet the study criteria (Figure 2). Among the remaining 197 persons, median age was 42 (range 1–81) years, most (119/197; 60%) were female, and 11 (6%) were pregnant (Table). Median length of travel was 11 (range 2–177) days. The diagnosis of recent Zika virus infection was made by serologic testing for 134 (68%) persons, by molecular testing for 57 (29%), and by molecular and serologic testing for 6 (3%).

**Table:** Demographics, travel data, and laboratory testing results for Zika virus disease patients, United States, January 1, 2015, through June 23, 2016

Patient characteristic	All cases, no. (%), n = 197*	Confirmed cases, no. (%), n = 79†
Age, y		
0–19	19 (10)	10 (13)
20–39	71 (36)	29 (37)
40–59	79 (40)	32 (40)
>60	27 (14)	7 (9)
Unknown	1 (<1)	1 (1)
Sex		
M	77 (39)	26 (33)
F	119 (60)	52 (66)
Unknown	1 (1)	1 (1)
Pregnant		
Yes	11 (6)	2 (3)
No	161 (82)	60 (76)
Unknown	25 (13)	17 (22)
Travel duration, d		
<7	24 (12)	15 (19)
7–13	88 (45)	64 (81)
14–20	31 (16)	0
21–27	12 (6)	0
≥28	42 (21)	0

*Persons with Zika virus–like symptoms and positive results for Zika virus RNA by real-time reverse transcription PCR or positive results for Zika or dengue virus IgM and Zika virus plaque reduction neutralization test (PRNT) results >10 and Zika virus titer >4-fold higher than dengue virus titer. †Persons who traveled <2 weeks, experienced Zika virus–like symptoms, and had positive Zika virus RNA results by real-time reverse transcription PCR or positive Zika or dengue virus IgM results and PRNT >10 and dengue PRNT <10.

The Weibull distribution fit our data best (parameter estimates in Technical Appendix Table 1). For the primary case set, our estimates for incubation period were median 6.2 (95% CI 5.7––6.6) days (Figure 1, panel B) and mean 6.4 (95% CI 5.7–7.0) days. We estimated that, among persons in whom symptoms would develop, they would develop in 5% by 2.1 (95% CI 1.7–2.4) days and in 99% by 13.6 (95% CI 13.0–14.2) days (Figure 1, panel B; Technical Appendix Table 2).

Of the 112 (57%) persons who had traveled for <2 weeks, cases were confirmed for 79 (71%). The age and sex distributions for these patients did not differ significantly from those of the primary case set (p = 0.67 and 0.44, respectively) (Table). The median length of travel was 8 (range 3–13) days. Zika virus diagnosis was confirmed by serologic testing for 47 (59%) patients, by molecular testing for 31 (39%), and by both methods for 1 (1%).

For patients with confirmed cases, we estimated the median incubation period to be 5.8 (95% CI 5.0–6.7) days (Figure 1, panel B; Technical Appendix Table 2) and the mean to be 6.0 (95% CI 5.2–6.8) days. The quantile estimates (5%–95%) for these patients were similar to those for all travelers; however, among travelers with shorter travel durations and confirmed Zika virus infections, symptoms developed within 11.8 (95% CI 10.8 –12.9 days) days for 99%, compared with 13.6 days for all travelers.

On the basis of our analysis, we estimate that the incubation period for Zika virus is 3–14 days. We expect symptoms to develop within 1 week of infection for 50% and within 2 weeks for 99%. Our estimates for Zika virus incubation period are similar to those reported for other flaviviruses (*9**–**12*). The incubation period for Zika virus has been estimated by Lessler et al., who reported data from 25 patients with variable exposure and laboratory evidence of infection (*13*). Their estimated median incubation period was similar to ours, 5.9 days, but the upper limit from that study was 18 days, which is 6 and 7 days longer than our estimates for the primary and secondary case sets, respectively. The difference in the upper limit was probably the result of the lower number of cases and higher variability in travel durations for their cohort.

Our analysis has several limitations. First, samples were submitted to the Centers for Disease Control and Prevention for all patients in this analysis, although guidance for testing recommended testing only persons with symptom onset <2 weeks after travel (*14*). Testing of all patients could have biased our sample population. Second, we included persons who were Zika virus IgM positive, considered as having recent infection. However, because the duration of IgM after Zika virus infection is not known, we might have included persons who had a prior infection unrelated to their most recent travel. Third, our analysis does not include other modes of transmission, such as sexual or congenital, for which incubation periods might differ. Fourth, we cannot be sure that all cases included in the analysis were caused by vector transmission because sexual transmission may have occurred during travel. Similarly, our primary case set included 11 pregnant women. Data suggest that the immunologic response to Zika virus infection might differ during pregnancy (*15*); however, in our analysis, the incubation periods of the pregnant women did not differ qualitatively from those of nonpregnant travelers.

## Conclusions

According to our analysis, among Zika virus–infected travelers who will become symptomatic, 99% will experience symptoms within 2 weeks of exposure and 50% within 1 week. Persons for whom symptoms develop >2 weeks after travel and test results for a recent Zika virus infection are positive should be evaluated for alternative modes of transmission (e.g., sexual transmission) or local vectorborne transmission.

Technical Appendix Parameter and incubation period quantile estimates for persons with confirmed Zika virus disease and for all persons with Zika virus disease, United States, January 1, 2015, through June 23, 2016.
