# Supplementary material for: Estimated Incubation Period for Zika Virus Disease
Source: Emerg Infect Dis. 2017 May;23(5):841–4. doi: 10.3201/eid2305.161715 (PMC5403043; doi:10.3201/eid2305.161715)
Supplement: Technical Appendix — Parameter and incubation period quantile estimates for persons with confirmed Zika virus disease and for all persons with Zika virus disease, United States, January 1, 2015, through June 23, 2016. [file 16-1715-Techapp-s1.pdf]

Article DOI: http://dx.doi.org/10.3201/eid2305.161715

Estimated Incubation Period for Zika Virus Disease

Technical Appendix

Technical Appendix Table 1. Parameter estimates (95% confidence intervals) for the fitted Weibull probability models detailed in (*1*) for both the primary and confirmed case sets

Case set	Shape parameter	Scale parameter
All Zika Virus Disease Cases*	2.39 (1.89, 2.88)	7.19 (6.48, 7.91)
Zika Virus Disease Confirmed Cases†	2.69 (1.83, 3.55)	6.70 (5.88, 7.52)

*All Zika virus disease cases samples are positive for Zika virus RNA by rRT-PCR or were Zika or dengue IgM positive and had a Zika PRNT ≥10 and 4-fold or higher Zika virus titer than dengue.
†Zika virus disease confirmed samples are positive for Zika virus RNA by rRT-PCR or were Zika or dengue IgM positive and had a Zika PRNT ≥10 and dengue PRNT <10.

Technical Appendix Table 2. Incubation period quantile estimates for individuals with Zika virus-like symptoms and positive test results for Zika virus infection by length of travel and test results

Case set	No.	5% (95% CI)	25% (95% CI)	50% (95% CI)	75% (95% CI)	95% (95% CI)	99% (95% CI)
All Zika Virus Disease Cases*	197	2.1 (1.7, 2.4)	4.3 (3.8, 4.7)	6.2 (5.7, 6.6)	8.2 (7.7, 8.8)	11.4 (10.8, 12.0)	13.6 (13, 14.2)
Zika Virus Disease Confirmed Cases†	79	2.2 (1.6, 2.8)	4.2 (3.5, 4.9)	5.8 (5.0, 6.7)	7.6 (6.7, 8.5)	10.1 (9.1, 11.1)	11.8 (10.8, 12.9)

*All Zika virus disease cases samples are positive for Zika virus RNA by rRT-PCR or were Zika or dengue IgM positive and had a Zika PRNT ≥10 and 4-fold or higher Zika virus titer than dengue.
†Zika virus disease confirmed samples are positive for Zika virus RNA by rRT-PCR or were Zika or dengue IgM positive and had a Zika PRNT ≥10 and dengue PRNT <10.

Reference

1. Reich NG, Lessler J, Cummings DAT, Brookmeyer R. Estimating incubation period distributions with coarse data. Stat Med. 2009;28:2769–84. PubMed http://dx.doi.org/10.1002/sim.3659
